# A safe, stable, and convenient three-dimensional device for high Le Fort I osteotomy

**DOI:** 10.1186/s40902-020-00276-1

**Published:** 2020-09-11

**Authors:** Keisuke Sugahara, Masahide Koyachi, Kento Odaka, Satoru Matsunaga, Akira Katakura

**Affiliations:** 1 Department of Oral Pathobiological Science and Surgery, Tokyo Dental College, 2-9-18 Kanda Misaki-cho, Chiyoda-ku, Tokyo, Japan; 2 Oral Health Science Center, Tokyo Dental College, 2-9-18 Kanda Misaki-cho, Chiyoda-ku, Tokyo, Japan; 3 Department of Oral and Maxillofacial Radiology, Tokyo Dental College, 2-9-18 Kanda Misaki-cho, Chiyoda-ku, Tokyo, Japan; 4 Department of Anatomy, Tokyo Dental College, 2-9-18 Kanda Misaki-cho, Chiyoda-ku, Tokyo, Japan

**Keywords:** 3D device, Le Fort I osteotomy, Orthognathic surgery

## Abstract

**Background:**

Le Fort I osteotomy is a highly effective treatment for skeletal jaw deformities and is commonly performed. High Le Fort I osteotomy is a modified surgical procedure performed for improving the depression of the cheeks by setting the osteotomy higher than the conventional Le Fort I osteotomy. Developments in three-dimensional (3D) technology have popularized the use of 3D printers in various institutions, especially in orthognathic surgeries. In this study, we report a safe and inexpensive method of performing a high Le Fort I osteotomy using a novel 3D device and piezosurgery, which prevent tooth root injury without disturbing the operation field for patients with a short midface and long tooth roots.

**Results:**

A 17-year-old woman presented with facial asymmetry, mandibular protrusion, a short midface, and long tooth roots. We planned high Le Fort I osteotomy and bilateral sagittal split ramus osteotomy. Prevention of damage to the roots of the teeth and the infraorbital nerve and accurate determination of the posterior osteotomy line were crucial for clinical success. Le Fort I osteotomy using 3D devices has been reported previously but were particularly large in size for this case. Additionally, setting the fixing screw of the device was difficult, because of the risk of damage to the roots of the teeth. Therefore, a different surgical technique, other than the conventional Le Fort I osteotomy and 3D device, was required. The left and right parts of the 3D device were fabricated separately, to prevent any interference in the surgical field. Further, the 3D device was designed to accurately cover the bone surface from the piriform aperture to the infra-zygomatic crest with two fixation points (the anterior nasal spine and the piriform aperture), which ensured stabilization of the 3D device. The device is thin and does not interfere with the surgical field. Safe and accurate surgical performance is possible using this device and piezosurgery. The roots of the teeth and the infraorbital nerve were unharmed during the surgery.

**Conclusions:**

This device is considerably smaller than conventional devices and is a simple, low-cost, and efficient method for performing accurate high Le Fort I osteotomy.

## Background

The first description of a Le Fort I surgery was in the German language by Lamgenbeck in 1859 and in the USA by Cheever in 1864 for the resection of a nasopharyngeal tumor. This procedure was used to correct an open bite from a Guerin-type fracture in 1927, when Wassmund repositioned the maxilla without separating it from the pterygoid processes. Obwegeser developed the modern Le Fort I osteotomy procedure in which he completely immobilized the maxilla with the pterygomaxillary disjunction [1]. Conventional Le Fort I osteotomy can be successfully performed for patients with isolated maxillary hypoplasia, and the high Le Fort I osteotomy can be performed for patients with midfacial hypoplasia and a pronounced zygomatic deficiency. High Le Fort I osteotomy is a modified surgical procedure performed for improving the depression of the cheeks by setting the osteotomy higher than the conventional Le Fort I osteotomy [2]. The osteotomy line includes a part of the zygoma.

Developments in three-dimensional (3D) technology over the past decade have popularized the use of 3D printers in several institutions, especially in orthognathic surgeries. The “Fab Lab TDC” was the first digital fabrication laboratory for dentistry in Japan, which was established in 2013 [3]. Various 3D devices have been reported previously [3–5].

Here, we report a safe and inexpensive method of performing high Le Fort I osteotomy using a novel 3D device and piezosurgery.

## Materials and methods

### Patient

A 17-year-old woman presented with facial asymmetry, mandibular protrusion, short midface, and long tooth. We planned a high Le Fort I osteotomy and bilateral sagittal split ramus osteotomy.

### Method

**Fig. 1:**
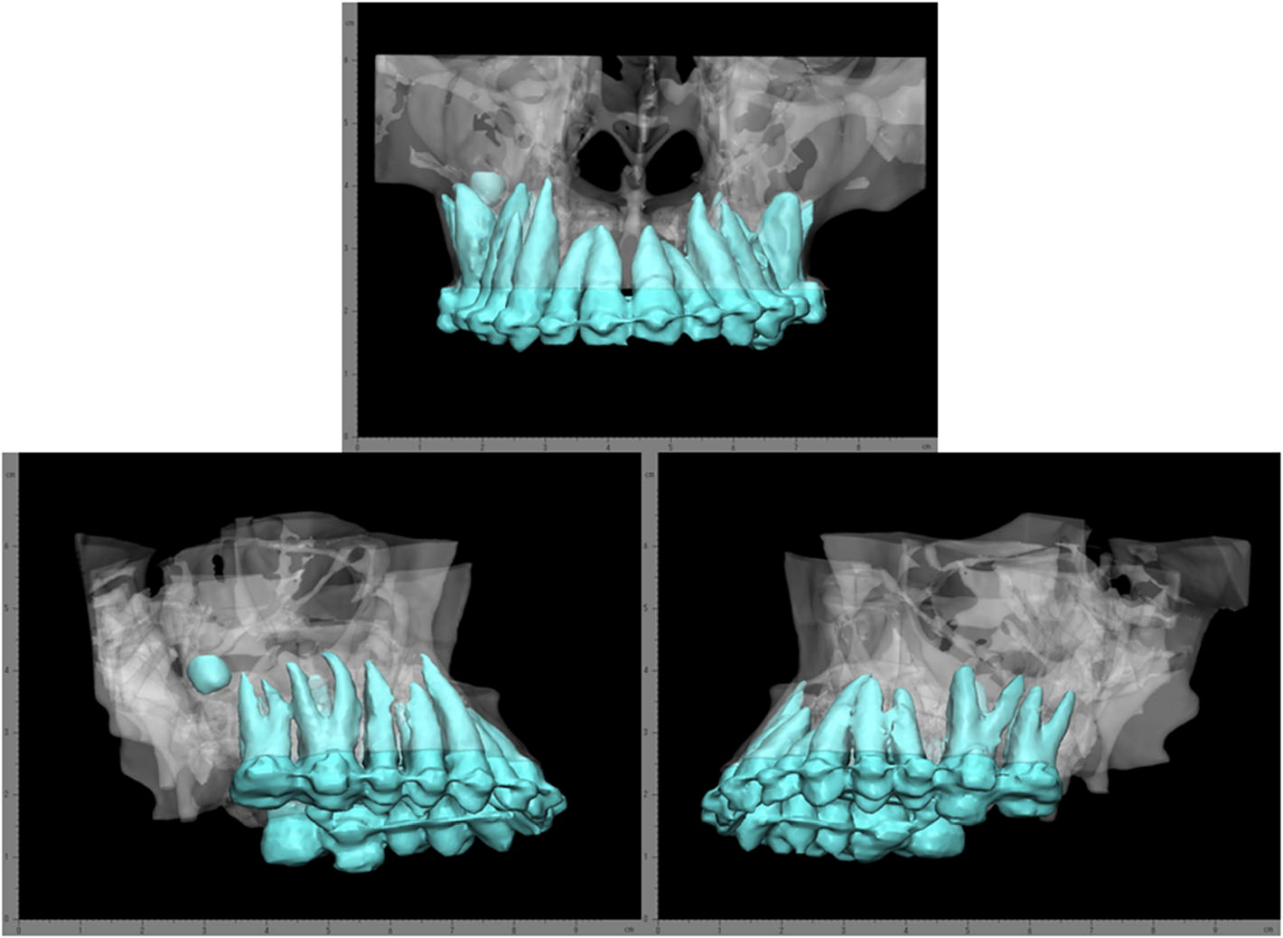
Three-dimensionally reconstructed data showing the presence of long tooth roots and right maxillary third molar. (Front, Right, Left side view)

We designed the 3D device of the high Le Fort I osteotomy to prevent tooth root injury without disturbing the operation field for patients with a short midface and long tooth roots (Fig. 1).

## Results

Prevention of damage to the roots of the teeth and the infraorbital nerve and accurate determination of the posterior osteotomy line was crucial for clinical success of the surgery. A method for accurate reproduction of virtually planned operations in Le Fort I osteotomy using 3D devices has been reported previously [5]. These 3D devices were stable but were particularly large in size for this case. Additionally, setting the fixing screw of the device was difficult, because of the risk of damage to the roots of the teeth. Therefore, a different surgical technique, other than the conventional Le Fort I osteotomy and 3D device, was required.

**Fig. 2:**
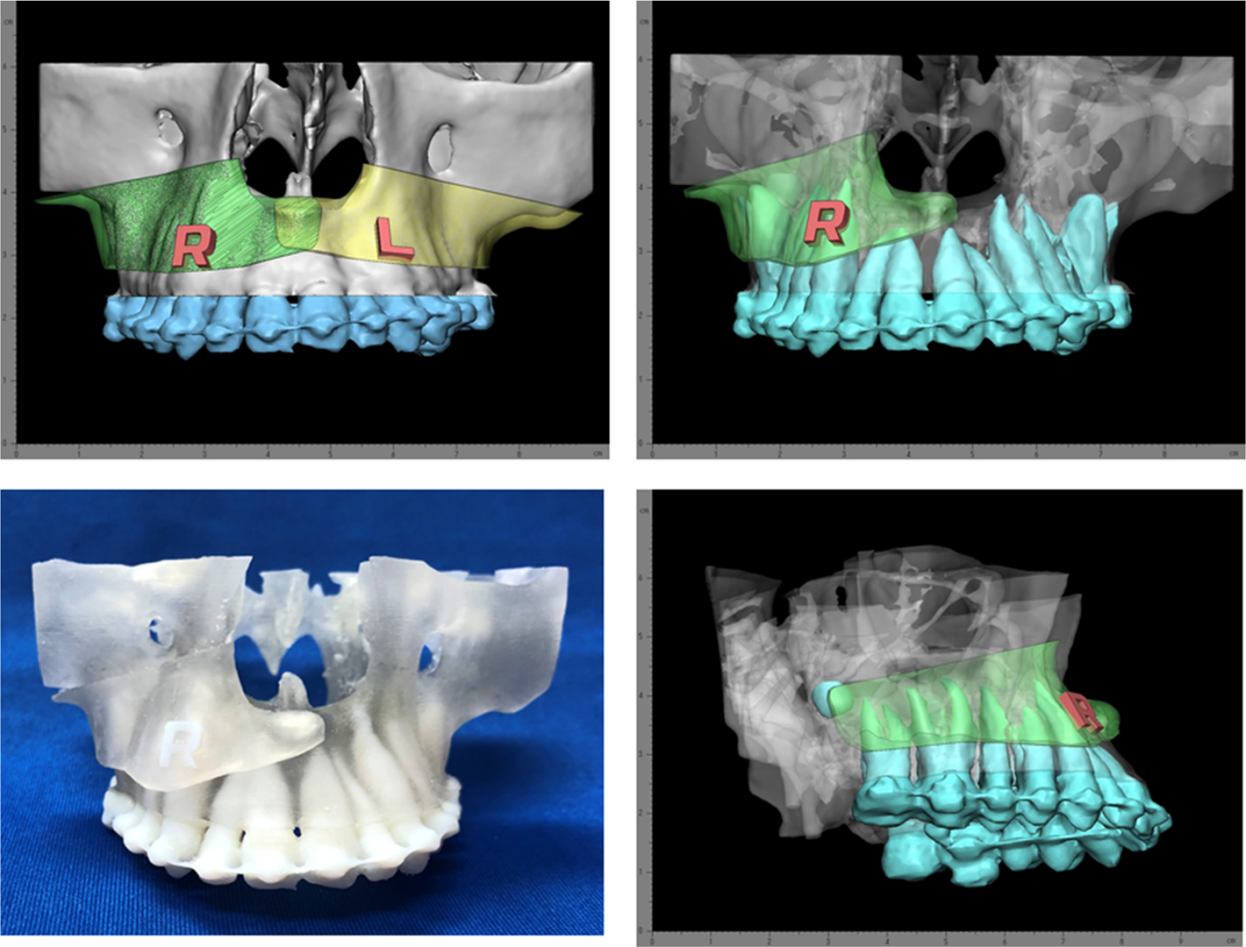
Design of the three-dimensional device, which covers the anterior nasal spine and piriform aperture. The left and right parts of the 3D device were fabricated separately. We made a 3D model to verify the devices fit

The computed tomography (CT) images of the patient were formatted as DICOM data, which were transferred to Minimics (Materialise, Leuven, Belgium) and 3-matic (Materialise, Leuven, Belgium), and set to allow the virtual operation. The left and right parts of the 3D device were fabricated separately, to prevent any interference in the surgical field. Further, the 3D device was designed to accurately cover the bone surface from the piriform aperture to the infra-zygomatic crest with two fixation points (the anterior nasal spine and the piriform aperture), which ensured stabilization of the 3D device (Fig. 2). All data were converted to STL format, and the 3D device was fabricated using a 3D printer (Objet 260 Connex, Stratasys Ltd., Minnesota) in the Fab Lab TDC.

**Fig. 3:**
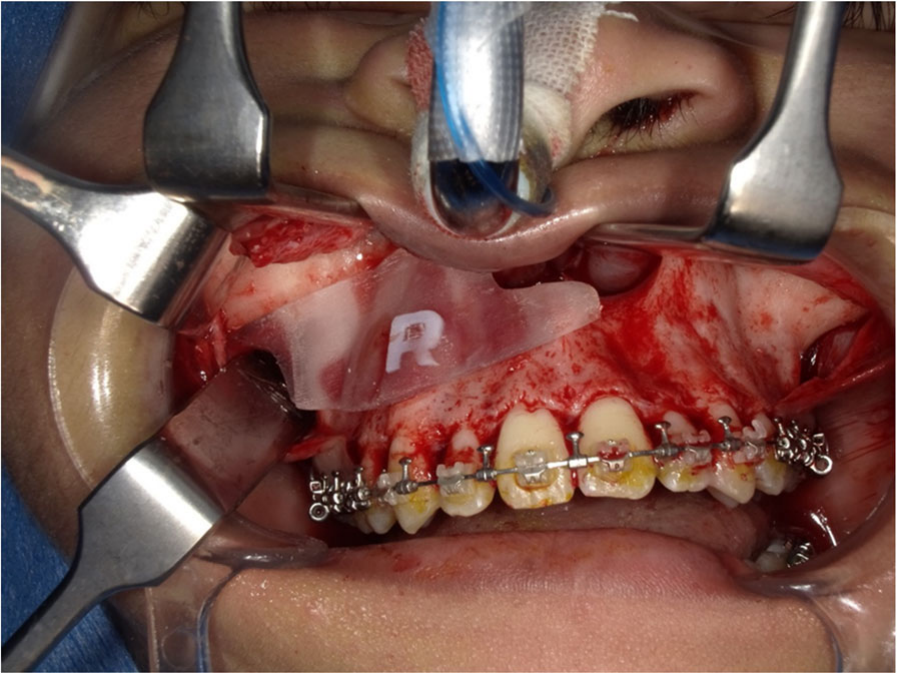
Intraoperative view of the osteotomy using the three-dimensional device (right side)

**Fig. 4:**
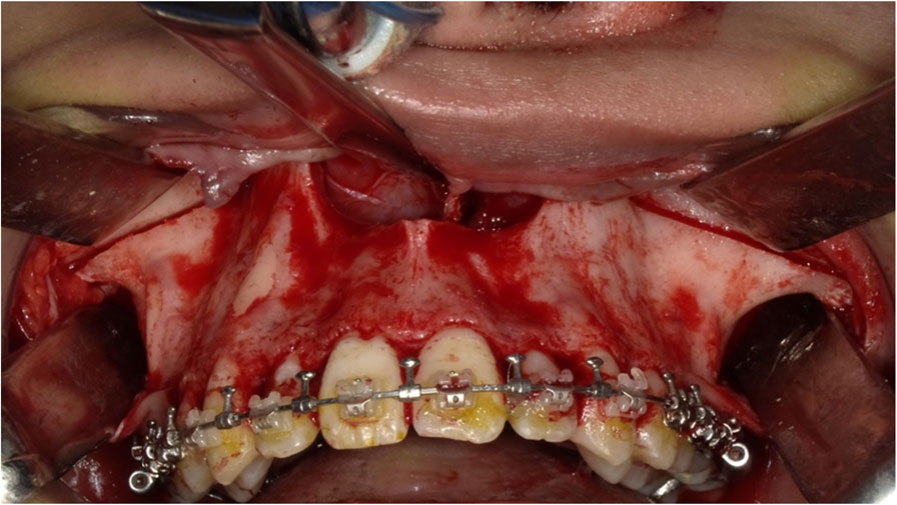
Intraoperative view after the osteotomy. The roots of teeth are unharmed

We chose the Neumann incision for this case, as it provides a sufficient field of view for the Le Fort I osteotomy; however, it leads to scarring in the maxillary vestibule (Figs. 3 and 4). Stability of the device during the procedure was ensured by maintaining complete contact of the 3D device with the bone surface instead of using a lateral incision.

## Discussion

The 3D printing method is primarily derived from an additive manufacturing technology. 3D printing seems to have various applications in oral and maxillofacial surgery, particularly since the release of general use 3D printers on the market several years ago. 3D printing techniques have been used for corrective osteotomies, including orthognathic surgery, and have achieved some promising results in the last decade [6].

Globally, customized metal plates for jaw deformation and reconstruction are made with CAD/CAM based on preoperative computer simulations [7–9]. However, in Japan, only premade plates can be used in jaw deformity patients. As first fabrication laboratory for dentistry in Japan was established at Tokyo Dental College—the “Fab Lab TDC”—in December 2013 [1]. Techniques to construct full-scale 3D models, such as of the jaw, based on computed tomography (CT) and magnetic resonance imaging (MRI) modalities have been reported recently [3–6].

We have also created preoperative 3D-printed models of cases for tumors in maxilla and mandible jaw deformities and used them primarily for consultations with patients and for preoperative simulations [3–5]. A method for accurate reproduction of virtually planned operations in the Le Fort I osteotomy using 3D devices has been reported previously [5]. These devices were stable but were large in size for this case. In addition, setting the fixing screw of the device was difficult, because of the risk of damage to the roots of the teeth. Therefore, a different surgical technique, other than the conventional Le Fort I osteotomy and 3D device, was required.

In the present study, we demonstrated that 3D devices manufactured using our design can be applied to perform safe, stable, and low-cost surgeries without the need for the fixing screw in patients with a short midface.

## Conclusion

The device is thin and does not interfere with the surgical field. Safe and accurate performance of the surgery is possible using this device and piezosurgery. The roots of the teeth and infraorbital nerve were unharmed during the surgery.

This device is very economical and is a simple and efficient method for accurate Le Fort I osteotomy.

## Data Availability

Not applicable

## Abbreviations

3D: Three-dimensional
CAD/CAM: Computer aided design/computer aided manufacturing
CT: Computed tomography
